# A Proposal for a Rat Model of Spinal Cord Injury Featuring the Rubrospinal Tract and its Contributions to Locomotion and Skilled Hand Movement

**DOI:** 10.3389/fnins.2016.00005

**Published:** 2016-01-27

**Authors:** Renée Morris, Ian Q. Whishaw

**Affiliations:** ^1^Translational Neuroscience Facility, School of Medical Sciences, The University of New South Wales AustraliaSydney, NSW, Australia; ^2^Department of Neuroscience, Canadian Centre for Behavioural Neuroscience, University of LethbridgeLethbridge, AB, Canada

**Keywords:** rubrospinal tract, skilled reaching, spinal cord injury, spinal cord repair, arpeggio

## Abstract

Spinal cord injury and repair is a dynamic field of research. The development of reliable animal models of traumatic spinal cord injury has been invaluable in providing a wealth of information regarding the pathological consequences and recovery potential of this condition. A number of injury models have been instrumental in the elaboration and the validation of therapeutic interventions aimed at reversing this once thought permanent condition. In general, the study of spinal cord injury and repair is made difficult by both its anatomical complexity and the complexity of the behavior it mediates. In this perspective paper, we suggest a new model for spinal cord investigation that simplifies problems related to both the functional and anatomical complexity of the spinal cord. We begin by reviewing and contrasting some of the most common animal models used for investigating spinal cord dysfunction. We then consider two widely used models of spinal deficit-recovery, one involving the corticospinal tracts (CTS) and the other the rubrospinal tract (RST). We argue that the simplicity of the function of the RST makes it a useful model for studying the cord and its functional repair. We also reflect on two obstacles that have hindered progress in the pre-clinical field, delaying translation to the clinical setup. The first is recovery of function without reconnection of the transected descending fibers and the second is the use of behavioral paradigms that are not under the control of the descending fiber pathway under scrutiny.

## Introduction

The most commonly used injury paradigms for spinal cord injury are contusions and transections. Contusions are produced by controlled blunt force directed to a portion of the cord, whereas transections consist of selective cuts to all or a portion of the cord. An advantage of contusion methods is that they produce histologically graded and consistent trauma (Wrathall et al., 1985) with pathological outcomes that are similar to spinal cord injury in human patients. This allows quasi-direct comparison between the two species (Metz et al., 2000). Contusion models are useful in the characterization of the morphological (Reyes-Alva et al., 2013), behavioral (Basso et al., 1996; Redondo-Castro et al., 2013), and neurological response of the spinal cord to injury (Gale et al., 1985; Agrawal et al., 2010; Detloff et al., 2013). These models are also valuable for measuring the efficacy of strategies to counteract secondary cell death and inflammatory reactions (Wang et al., 2011; Andrews et al., 2012; Mountney et al., 2013). Contusion injuries leave intact a number of long ascending and descending fiber tracts, however, making them less amenable to investigations into the effect of therapeutic compounds on axonal regeneration.

Complete transection models are ideal for exploring axonal regeneration across the level of injury, as there is no issue of deciding between regenerating fibers and spared ones. The two ends of the transected spinal cord tend to retract away from each other, creating a fluid-filled cavitation that is not conducive to axonal regeneration (Steward et al., 2003). Researchers have taken advantage of this situation to introduce implants and bridges in the spinal cord cavity, thus creating an environment that is potentially conducive to axonal regeneration (e.g., García-Alías et al., 2011; Min et al., 2011; Ziegler et al., 2011; Choi et al., 2012; Kang et al., 2012; Aizawa-Kohama et al., 2013; Dai et al., 2013). Hemisection models reduce the amount of post-injury care required for the operated animals. One of the drawbacks of spinal cord hemisection is the difficulty in establishing evidence that the surgical approach has indeed severed all the axons, but this can be ascertained by tract-tracing techniques.

## Spinal cord transection destroys at least two fiber tract systems

**Figure 1:**
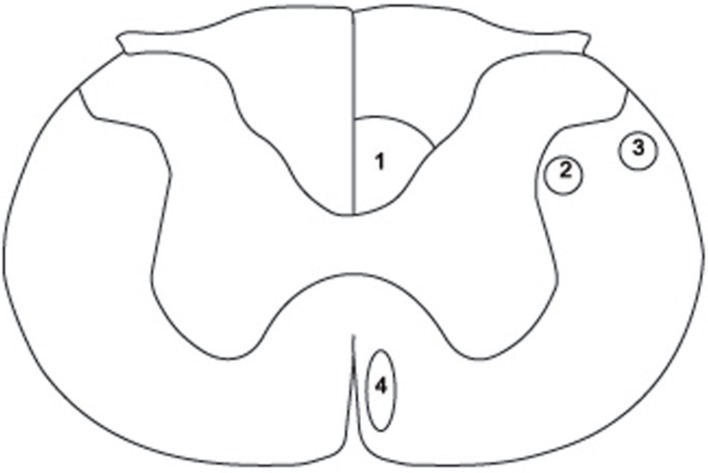
**Schematic diagram of a cross section through the rat spinal cord at cervical level 3 (C3) to illustrate the position of the three components of the corticospinal tract (CST) and the rubrospinal tract (RST)**. (1) dorsal CST, (2) lateral CST, (3) RST, (4) ventral CST. Adapted from Paxinos and Watson (2005).

Transection injury models, whether complete or partial (i.e., lateral and dorsal hemisections and lateral funiculus transection), damage at least two major descending fiber tract systems: the corticospinal tract (CST) and the rubrospinal tract (RST) (see Figure 1). Although in relatively close proximity, these two systems respond differently to therapy scenarios that aim to trigger axonal regeneration. For example, treatment with neurotrophin-3 (NT-3) has been shown to induce growth in the CST (Blits et al., 2000; Tuszynski et al., 2003; Hagg et al., 2005; Chen et al., 2006; Chen and Shine, 2013; Weishaupt et al., 2014), whereas brain-derived neurotrophic factor (BDNF) exerts its trophic effect mainly on the RST (Liu et al., 2002; Koda et al., 2004; Kwon et al., 2007; Bretzner et al., 2008; Conova Grous et al., 2013). Taken together, this evidence suggests that therapeutic intervention with a neurotrophic factor, either NT-3 or BDNF, is doomed to be ineffective in stimulating regeneration in at least one of the two descending motor pathways unless both neurotrophins are used conjointly.

There is also evidence suggesting that the RST and the CST in the rat make conjoint yet different contributions to skilled forelimb movement, reaching for and handling food items and locomotion (Whishaw et al., 1993, 1998; Metz et al., 1998; Muir and Whishaw, 1999; Hendriks et al., 2006; Muir et al., 2007; Kanagal and Muir, 2009). One can argue that an important shortcoming of the current models of spinal cord injury is that they lack functional specificity because of the broad, confounding anatomical and functional deficits that they create. They are difficult to interpret because the lesions are heterogeneous and the resulting behavioral deficits are complex. Therefore, a single fiber tract model of spinal cord injury provides a more elegant approach for the study of axonal regeneration than a transection model that is not selective. Indeed, a single fiber tract model allows the establishment of the precise contribution of the descending tract of interest in the control of movement. Such knowledge can thus be used to establish predictions as to the expected outcomes of effective therapeutic treatments. Here we compare the utility of CST and RST single tract models of spinal cord injury.

## Corticospinal tract models

Despite the wealth of behavioral studies investigating the function of the rat CST, a clear role for this tract is a matter of debate. Pyramidotomy at the level of the medulla abolishes movements that are elicited by intracortical microstimulation of the forelimb-associated region of the motor cortex in naïve animals (Piecharka et al., 2005). Pyramidotomy also significantly impairs proximal and distal movement of the forelimb (Whishaw et al., 1993, 1998; Whishaw and Metz, 2002). Nevertheless, it can be difficult to interpret these effects, as these transections also sever non-corticospinal connections. Further, transection of the spinal cord dorsal column at cervical segments C1/C2, in which runs the main component of the CST, spares a number of measures of skilled reaching (Alstermark and Pettersson, 2014). Unlike pyramidotomy, dorsal column transection leaves intact the reticulo-spinal pathway. The authors concluded that these findings rule out the contribution of the CST in skilled reaching and suggest that the cortico-reticulo-spinal pathway plays an important role in this motor behavior.

It is worth noting that transection of the dorsal column at cervical segments C1/C2 by Alstermark and Pettersson (2014) also leaves intact the ventral and lateral CST, as their fibers diverge from the main CST at the pyramidal decussation, i.e., rostral to C1/C2. This raises the possibility that the lack of impairment in skilled reaching could be due to the sparing of the lateral and/or the ventral aspect of the CST and not to the preservation of the reticulo-spinal tract. In this respect, we show that complete lesions of the lateral funiculus, in which the lateral CST and the RST run, impair two movement elements of skilled reaching, namely arpeggio and grasping (Morris et al., 2011). The arpeggio movement consists of digit opening and a lateral-to-medial pronation of the hand to grasp. Grasping consists of an in place flexing and closing of the digits and dorsiflexion of the wrist to capture the target. Further, we present evidence that lesions that selectively disrupt the RST only abolished the arpeggio movement, suggesting that the lateral CST plays a role in the movement element of grasping.

Data obtained with transgenic labeling of the CST have revealed the presence of two distinct populations of axons within the mouse CST. Although the majority of fibers running within the CST are thin, with diameters ranging from 0.4 to 0.6 μm, the ventral and lateral contingents of CST fibers are also populated by heavily myelinated axons with diameters of 1.5–5 μm (Bareyre et al., 2005). It has also been demonstrated that transection of the dorsal CST resulted in a loss of 80–97% of the fiber projections to the dorsal and intermediate spinal cord laminae, while leaving intact the projections to the ventral horn where motor neurons are located. These findings suggest that, although most CST axonal projections originate from its dorsal aspect, the direct, descending input to the ventral horn is derived from its minor components (see Steward et al., 2004). One can speculate that the rodent CST is functionally segregated. A contingent of numerous but small, poorly myelinated fibers within the dorsal CST innervate the spinal cord motor neurons indirectly, i.e., via synaptic contact with relay interneurons located in the intermediate laminae of the cord. A contingent of sparse but large, fast-conducting axons within the lateral CST perhaps provides input more directly to motor neurons. It is interesting to note that the lateral CST in rodents is found in the same location as the primate main CST that forms monosynaptic contact with motor neurons supplying the forelimb and hindlimb. It would be interesting to investigate whether the rodent and primate lateral CST are homologous.

What is the unique function of the dorsal CST? Interestingly, dorsal (but not lateral) CST transection permanently abolishes the down conditioning of the H-reflex in the rat, whereas the ablation of the lateral column has no effect on this type of operant conditioning (Chen and Wolpaw, 1997; Chen et al., 2006). In this paradigm, rats, mice, monkeys, and humans learn to gradually decrease the amplitude of their H-reflex, i.e., the electrical analog of the spinal stretch reflex, in response to a reward contingency (Chen et al., 1999). The control of the H-reflex by the dorsal CST is in line with the anatomical observation that, in rodents, this component of the CST is two synapses away from the motor neurons involved in this reflex. Excitatory input from the dorsal CST on interneurons subsequently provides an inhibitory input to the motor neurons that consequently diminishes the amplitude of the H-reflex. The anatomy also explains why the destruction of putative direct descending input onto motor neurons, such as that provided by the lateral CST, would not interfere with the down conditioning of the reflex. Furthermore, the fact that strokes over the sensorimotor cortex interfere with spinal stretch reflex conditioning in humans brings translational significance to this simple test (Segal, 1997). H-reflex down conditioning could therefore be used to measure deficits and recovery after dorsal CST transection and therapeutic regimens.

## Rubrospinal tract models

The RST travels as a single bundle of axons within the dorsolateral funiculus of the spinal cord. The majority of the RST fibers terminate into the dorsal horn and in the intermediate region of the ventral horn (Brown, 1974). There are also functional monosynaptic connections between the RST and spinal cord motor neurons. For instance, when injected in forelimb muscles, rabies virus retrogradely labels the motor neurons that supply these muscles and produces significant trans-neuronal labeling in the red nucleus (Ruigrok et al., 2008). Furthermore, low-threshold microstimulation of the rat red nucleus results in short-latency EMG responses in forelimb muscles that are accompanied by a strong extension of the wrist (Küchler et al., 2002).

Excitotoxic lesions to the red nucleus, i.e., the origin of the RST, do not interfere with endpoint measures of skilled reaching, such as the reaching itself or its success in obtaining food (Whishaw et al., 1990, 1992, 1998; Whishaw and Gorny, 1996). However, red nucleus lesions interfere with several components of the reaching action, including limb aiming, pronation, and supination of the paw (Whishaw and Gorny, 1996; Whishaw et al., 1998). Red nucleus lesions also abolish the arpeggio movement whereby the paw is pronated so that each digit (i.e., digits 5–2) sequentially makes contact with the shelf where the food is located. Transection of the lateral funiculus at cervical levels, a surgical procedure that ablates the RST, also interferes with several elements of the reach, including digit flexion and grasping (Schrimsher and Reier, 1993), supination and arpeggio (Muir et al., 2007; Kanagal and Muir, 2009), the advance of the limb toward the food target, the opening of the digits, and pronation and supination movements around the wrist (Stackhouse et al., 2008). It is worth noting, however, that the lesions performed in these studies encompassed several other ascending and descending fiber tracts than the RST, potentially accounting, at least partly, for the wide range of behavioral deficits that they produce.

We have used a behavioral/anatomical fractionation method to isolate the behavioral contribution of the RST. This method reveals that unilateral lesions that specifically disrupt the RST at cervical levels have a relatively selective effect on the forelimb movements used in reaching for food (Morris et al., 2011). Normally, as the reaching hand of a rat approaches a food target, it is pronated to grasp the food. Pronation is distinctive in that the fingers, which are extended when the limb is advanced, are opened gradually through pronation in a lateral to medial topography. This movement is termed *arpeggio* because it is similar to the movement of the fingers of a piano player in performing an arpeggio. After RST lesions, the rat's arpeggio movement is disrupted while leaving other movement elements of the reaching action largely intact (Morris et al., 2011). The contribution of the RST in the control of the arpeggio movement is supported by recent findings that a lesion to the magnocellular subdivision of the red nucleus, from which the RST specifically arises, also disrupts this movement (Morris et al., 2015). Moreover, reports that lesions to the CST have no deleterious effect on the arpeggio movement support the unique involvement of the RST in the execution of this movement (Whishaw et al., 1998; Kanagal and Muir, 2009).

It is interesting that the arpeggio movement is also featured in forelimb stepping on the rotarod walking apparatus (Whishaw et al., 2008) as well as in overground walking where rats stride on an elevated alley in order to reach a home cage (Whishaw et al., 2010). Indeed, after the limb is advanced forward to complete a stride, digit 5 is the first digit to contact the floor or the drum surface, after which digits 4–2 sequentially make contact with the surface in an arpeggio movement. We have preliminary evidence that RST lesions or lesions to the magnocellular subdivision of the red nucleus abolish the arpeggio movement during forelimb stepping as a rat moves forward from the back of the reaching box toward the shelf as a new trial is generated (see Movie 1). The integrity of the RST is therefore critical for limb use in both reaching for food and walking (Hendriks et al., 2006; Muir et al., 2007; Kanagal and Muir, 2009). In contrast, CST lesions do not impair spontaneous or skilled locomotor activity (Metz et al., 1998; Muir and Whishaw, 1999; Loy et al., 2002; Metz and Whishaw, 2002; Kanagal and Muir, 2009). It is worth mentioning, however, that rats are able to generate basic stepping even after the removal of all supraspinal input to the spinal cord (Zhang et al., 2007). Taken together, the anatomical and behavioral evidence suggests that that the RST model commends itself to spinal cord investigations. Damage to the RST seems to selectively affect the arpeggio movement in both locomotion and skilled movements. Thus, this specific transection-functional model can be used as a powerful behavioral readout against which the success of a given repair strategy can be validated.

## Obstacles to RST functional regeneration after treatment

So far, the field of recovery of function has focused on developing therapeutic strategies that trigger axonal elongation or sprouting and that lead to improved motor function. In this context, there is accumulating evidence that treatment with BDNF after lesions that damage the RST trigger some axonal regeneration that is accompanied by various degrees of amelioration of motor performance (Liu et al., 1999; Namiki et al., 2000; Kim et al., 2001; Blits et al., 2003; Shumsky et al., 2003; Koda et al., 2004; Ruitenberg et al., 2004; Tobias et al., 2005). For neuroanatomists, the report of recovery of function without reconnection of the transected descending fibers on their post-synaptic targets (i.e., motor neurons below the level of the lesion) is perplexing. How can such treatments ameliorate motor performance if they do not re-establish innervation of the RST onto motor neurons?

It is clear that the delivery of BDNF to the injured spinal cord creates a growth-permissive environment for the RST that has the potential to offset the deleterious effect of inhibitory molecules that act as a barrier for axonal regeneration (reviewed in Morris, 2014). Further, BDNF-secreted cells implanted in the injured spinal cord contribute to reducing cavity and scar formation (Ramer et al., 2004), assist in the sparing of myelin (Nakajima et al., 2012), reduce lesion volumes (Alexanian et al., 2011; Walker and Xu, 2014), decrease inflammatory responses (Abrams et al., 2009; Nakajima et al., 2012; Jia et al., 2014; Zhao et al., 2014), and provide neuronal and non-neuronal protection (Sasaki et al., 2009; Walker and Xu, 2014). Taken together, these data suggest that treatments that ameliorate motor performance without reconnecting the damaged axons with their former post-synaptic targets do so mainly by diminishing the deleterious effects of the secondary injury. Sprouting of the transected descending tracts that could activate local spinal circuits is also suggested to play a role in motor function recovery after spinal cord injury, however more work needs to be carried out in order to instantiate this view.

Several behavioral paradigms have been used to measure the therapeutic benefits of BDNF treatment after lesions that destroy the RST. These include Schallert's cylinder test (Liu et al., 1999; Shumsky et al., 2003; Tobias et al., 2005), the horizontal rope crossing test (Kim et al., 2001; Shumsky et al., 2003; Ruitenberg et al., 2004; Tobias et al., 2005), the open-field locomotor rating scale (BBB; Kim et al., 2001; Blits et al., 2003; Shumsky et al., 2003; Koda et al., 2004; Tobias et al., 2005), the narrow beam walking test (Kim et al., 2001; Shumsky et al., 2003), and the swim test (Kim et al., 2001). Overall, these tests mainly measure limb use *per se* in relation to the intact limb(s) and locomotor capacity. We have demonstrated that lesions that affect the integrity of the RST or of its cells of origin in the red nucleus do not produce deficits in whole-limb movement (Whishaw et al., 1998; Morris et al., 2011, 2015). Furthermore, cervical bilateral lesions of the dorsolateral funiculus in which the RST runs do not impair spontaneous forelimb use, as measured by the cylinder test (Muir et al., 2007). It is our opinion that an effective treatment that involves the functional restoration of the RST would result in the return of the arpeggio movement in reaching and walking.

## Conclusion

Progress in the field of spinal cord injury and repair has been hampered by the naïve view that axonal elongation without reconnection with former post-synaptic targets could lead to the recovery of function. Furthermore, advances in the field have been hindered by the use of behavioral paradigms that are not under the control of the descending fiber pathway under scrutiny and that lack translational relevance. The latter obstacle can be eliminated by the use of behavioral tests that can be used in clinical settings. In this regard, the use of tests of skilled walking and reaching, although more time consuming, offer tools to generalize findings obtained in animal models to the clinic. Indeed, the movements used by rats and humans to walk and reach are similar, especially with respect to lateral medial pronation of the limb (Sacrey et al., 2009; Klein et al., 2012). As a result, the movement elements of the reaching action can be evaluated with the same movement scale in the two species, therefore allowing clinicians working with spinal cord-injured patients to draw invaluable information from pre-clinical investigations.

## Funding

This work has been supported by a National Health and Medical Research Council of Australia Project Grant (RG122619) to RM.

### Conflict of interest statement

The authors declare that the research was conducted in the absence of any commercial or financial relationships that could be construed as a potential conflict of interest.
