# Supplementary material for: A Proposal for a Rat Model of Spinal Cord Injury Featuring the Rubrospinal Tract and its Contributions to Locomotion and Skilled Hand Movement
Source: Front Neurosci. 2016 Jan 27;10:5. doi: 10.3389/fnins.2016.00005 (PMC4728831; doi:10.3389/fnins.2016.00005)
Supplement: Supplementary file 2 [file DataSheet1.pdf]

**Legend**

Movie 1. Slow motion video recording of a rat walking forward and reaching for food before (A) and after (B) a lesion to the magnocellular subdivision of the red nucleus from which the RST originates. In (A), as the rat reaches the food pellet, digit 5 is the first digit to contact the shelf where the food is located, after which digits 4 to 2 sequentially make contact with the shelf so that the hand is fully pronated (wrist positioned parallel to the shelf) and all the fingers touch the shelf before grasping the food. Similarly, as the rat walk towards the shelf, digit 5 is the first digit to contact the floor, followed by digits 4, 3 and 2 in an arpeggio movement. In (B), while the rat is reaching, digit 5 is the first to contact the shelf but the arpeggio does not take place. Instead, the hand remains supinated (wrist positioned obliquely with regard to the shelf) as digits 4 and sometimes digit 3 contacting the shelf. As the rat with the lesion walks towards the shelf no lateral to medial pronation takes place. Instead, the rat's affected limb is 'flat-footed': the longest finger i.e., digit 3 touches the floor first. Then the intermediate fingers in terms of length i.e., digits 2 and 4 come in contact with the floor, followed by the shortest i.e., finger digit 5.
